# Supplementary material for: Distribution, prevalence of non-tuberculous mycobacteria on Hainan Island and antibiotic resistance of Mycobacterium abscessus
Source: Microbiol Spectr. 2025 Nov 28;14(1):e01134-25. doi: 10.1128/spectrum.01134-25 (PMC12772351; doi:10.1128/spectrum.01134-25)
Supplement: Supplemental figure legend — Figure S1 legend. [file spectrum.01134-25-s0001.docx]

**Supplemental Figure 1** Phylogenetic tree of hsp65 gene from 114 NTM using the neighbor-joining method with MEGA11.0 software. Tree-scale 0.009 represents the evolutionary distance unit, and Bootstrap values represents the self-expanding value, which is used to test the calculated evolutionary Tree branch confidence.
